# Genome-wide investigation and expression profiling of *APX* gene family in *Gossypium hirsutum* provide new insights in redox homeostasis maintenance during different fiber development stages

**DOI:** 10.1007/s00438-017-1413-2

**Published:** 2018-01-06

**Authors:** Chengcheng Tao, Xiang Jin, Liping Zhu, Quanliang Xie, Xuchu Wang, Hongbin Li

**Affiliations:** 1 College of Life Sciences, Key Laboratory of Agrobiotechnology, Shihezi University, Shihezi, Xinjiang China; 2 Institute of Tropical Biosciences and Biotechnology, Chinese Academy of Tropical Agricultural Sciences, Haikou, Hainan China

**Keywords:** Ascorbate peroxidase, Expression profiling, Redox homeostasis, *Gossypium hirsutum*, Cotton fiber

## Abstract

Ascorbate peroxidase (APX) is a member of heme-containing peroxidases which catalyze the H_2_O_2_-dependent oxidation of a wide range of substrates in plants and animals. As is known, H_2_O_2_ acts as a signaling molecule in the regulation of fiber development. Our previous work reported that ascorbate peroxidase 1 (*GhAPX1*) was important for cotton fiber elongation. However, knowledge about *APX* gene family members and their evolutionary and functional characteristics in cotton is limited. Here, we report 26 *GhAPX* genes by genome-wide investigation of tetraploid cotton *Gossypium hirsutum*. Phylogenetic and gene structure analyses classified these *APX* members into five clades and syntenic analysis suggested two duplication events. Expression profiling of the 26 *APXs* revealed that ten members are expressed in cotton fibers. Notably, *GhAPX10A, GhAPX10D, GhAPX12A*, and *GhAPX12D* showed high expression levels in 30-day fiber, while *GhAPX1A*/*D, GhAPX3A*/*D*, and *GhAPX6A*/*D* showed very low expression levels. The enzyme activity and H_2_O_2_ content assays revealed that cotton fiber kept high enzyme activity and the lowest H_2_O_2_ level in 30-day fibers, indicating that other than *GhAPX1*, the newly reported *APX* members are responsible for the reactive oxygen species homeostasis in the cotton fiber maturation stages. Expression profiling of ten fiber-expressed *APXs* after phytohormone treatments revealed their regulation patterns by different stimuli, suggesting that *GhAPX1, GhAPX12A*, and *GhAPX12D* are responsible to most phytohormone treatments. Our data provided evolutionary and functional information of *GhAPX* gene family members and revealed that different members are responsible to redox homeostasis during different cotton fiber development stages.

**Electronic supplementary material:**

The online version of this article (10.1007/s00438-017-1413-2) contains supplementary material, which is available to authorized users.

## Introduction

Tetraploid upland cotton (*Gossypium hirsutum*) is the most widely cultivated cotton plants, which is the most important resource of natural fiber for textile industry (Zhu 2016). The genome of tetraploid cotton (*G. hirsutum*, AADD, 2*n* = 4*x* = 52) and its diploid ancestor cotton (*Gossypium arboretum*, AA, 2*n* = 2*x* = 26; *Gossypium raimondii*, DD, 2*n* = 2*x* = 26) had been sequenced recent years (Paterson et al. 2012; Wang et al. 2012; Li et al. 2014, 2015; Zhang et al. 2015). Benefit from the publication of the genome of upland cotton (70,478 predicted protein coding genes), a large number of functional genes and their gene family members involved in cotton fiber development have been reported (Huang et al. 2015; Guo et al. 2016; Zhang et al. 2016; Wu et al. 2017). However, in-depth digging of the genome data will be necessary.

Reactive oxygen species (ROS) can be continuously produced in all aerobic organisms to take important role as regulator for cellular response to environmental factors in plants (Alscher et al. 1997; Pandey et al. 2017). It is demonstrated that H_2_O_2_, a signaling molecule known as the major active ROS type, is involved in regulation of plant cell development and stress resistance, such as root hair initiation and elongation, drought and salinity resistance, and temperature stress response (Pei et al. 2000; Panchuk et al. 2005; Pinheiro and Chaves 2011; Qu et al. 2013). ROS could synergize or antagonize many cellular regulatory circuits through active interaction with other signals and plant hormones during growth, development, and stress responses (Petrov and Breusegem 2012; Tao et al. 2016). In plant cells, despite the vital role in cell development, ROS accumulation could also cause severe damages, accordingly, plants developed a complex antioxidant system to prevent cellular damage generated by ROS, such as ascorbic acid (AsA), glutathione (GSH), and carotenoids (Smirnoff 2000).

Ascorbate peroxidase (APX, EC 1.11.1.11) is a family of type I heme-containing peroxidase that catalyzes H_2_O_2_ to water using ascorbate as specific electron donor, functioning in maintaining cell reduction/oxidation (redox) homeostasis by scavenging ROS (Foyer and Halliwell 1976; Noctor and Foyer 1998; Sharp et al. 2003; Suzuki et al. 2012). APXs are encoded by small multigene families in higher plants and are classified into different groups according to their subcellular localization (Teixeira et al. 2004). In *Arabidopsis*, eight APXs have been identified with localization of three in cytosol, three in peroxisome, and two in chloroplast, respectively (Shigeoka et al. 2002; Chew et al. 2003). *APX* family has been fully characterized in rice and tomato based on their genomes (Teixeira et al. 2006; Najami et al. 2008).

Cotton fiber, composed of numerous non-branched single cell, is an ideal model material to investigate cell growth (Li et al. 2017). ROS promoted cell expansion or enlargement though participating in plant cell wall loosening (Cosgrove 2000; Liszkay et al. 2004). It has been demonstrated that ROS plays vital role in cotton fiber cell elongation development (Li et al. 2007; Qin et al. 2008; Mei et al. 2009). Previously, we reported that *GhAPX1* plays a significant role in cotton fiber elongation *via* involving in ethylene signaling pathway (Li et al. 2007). Over-expression of cotton *GhAPX1A*/*D* increased fiber resistance to H_2_O_2_ stress (Guo et al. 2016). However, detailed knowledge about the whole *APX* family and expression patterns of *APX* genes in *G. hirsutum* remains unclear.

In this work, we performed genome-wide investigation and expression profiling of *APX* family in *G. hirsutum*. A total of 26 *GhAPX* genes were identified. Phylogenetic and gene structure analyses classified these *APX* members into five clades and syntenic analysis suggested two duplication events. Expression profiling of the 26 *APXs* revealed that ten members are expressed in cotton fibers. Interestingly, *GhAPX10A, GhAPX10D, GhAPX12A*, and *GhAPX12D* showed high expression levels in 30-day fiber, while *GhAPX1A*/*D, GhAPX3A*/*D*, and *GhAPX6A*/*D* showed very low expression levels at the same development stage. The enzyme activity and H_2_O_2_ content assays revealed that cotton fiber kept high enzyme activity and the lowest H_2_O_2_ level in 30-day fibers, indicating that other than *GhAPX1*, the newly reported *APX* members are responsible for the reactive oxygen species homeostasis in the secondary cell wall biosynthesis and maturation of cotton fiber development stages. This work provided evolutionary and functional information of *GhAPX* gene family members and revealed that different *GhAPX* family members are responsible to redox homeostasis during different cotton fiber development stages.

## Materials and methods

### Plant growth and different treatments

Cotton plants (*G. hirsutum* L. cv. Xuzhou 142) and the *fuzzless-lintless* mutant (*fl*) were grown in an experimental field at the Institute of Tropical Biosciences and Biotechnology in Haikou, China. Cotton bolls were labeled on the day of anthesis (defined as 0d ) and then detached in different developmental stages (5d, 10d, 15d, 20d, 25d, and 30d). The 10d fibers were treated with 200 µm ethephon (ETH), 100 µm H_2_O_2_, 100 µm gibberellin (GA), 100 µm methyl jasmonate (MeJA), 100 µm brassinolide (BR), and 1 mg/L of indole-3-acetic acid (IAA) for 1, 3, 6, and 12 h, respectively (Xin et al. 2016). Different tissues of roots, stems, leaves, petals, anthers, ovules, and fibers were immediately frozen in liquid nitrogen, and then stored at − 80 °C after stripped and separated from each other.

### Identification and multiple alignment of GhAPXs

Genome data of *G. hirsutum* were downloaded from Cotton Genome Project (http://cgp.genomics.org.cn/page/species/index.jsp) and CottonGen database (https://www.cottongen.org/) as described (Li et al. 2015; Zhang et al. 2015). The APX sequences of *Arabidopsis, Oryza sativa*, and *Theobroma cacao* were used as seed sequences to obtain the cotton APXs by local BLASTP through searching with a cutoff *e* value of 1e−10. Obtained 26 GhAPXs were submitted to InterProScan (http://www.ebi.ac.uk/interpro/) to assess the APX domains (IPR002016). Multiple sequence alignment was performed using ClustalX (2.0) with default parameter (Larkin et al. 2007).

### Chromosomal location analysis and phylogenetic tree construction

MapInspect software was used to visualize the distribution of the 26 *APX* genes in *G. hirsutum* chromosomes. Phylogenetic tree was constructed using MEGA 5.1 software with neighbor-joining method and bootstrap values of 1000 replicates as described (Tamura et al. 2007).

### Intron–exon and motif structure analysis of *APX* family

Gene Structure Display Server (http://gsds.cbi.pku.edu.cn/index.php) was used to analyze the intron–exon structure by comparing the CDS of *APX* genes with their corresponding genomic sequences (Hu et al. 2015). Deduced protein sequences of GhAPXs were submitted to multiple expectation maximization for motif elicitation (MEME) program for the identification of the conserved motifs (Bailey et al. 2006).

### Syntenic and evolutionary analyses

Paralogous *GhAPX* gene pairs were estimated based on their nucleotide identities > 90%. Tandem duplication events occurred when two closely related *GhAPX* genes are located within the same chromosome region. Segmental duplication has been defined as paralogous genes. The syntenic relationships of paralogous and orthologous between cotton and a closely related cacao species were analyzed using Circos program (Krzywinski et al. 2009) based on sequence identity calculations and the phylogenetic tree.

Evolutionary analyses were performed as previously reported (Jin et al. 2017). Briefly, the *Ka* (nonsynonymous substitution rate) and *Ks* (synonymous substitution rate) were calculated by DnaSP 5.0 software. The *Ka*/*Ks* ratios for *GhAPX* genes were used to assess the selection pressure on duplicated genes and *Ka*/*Ks* ratio > 1, < 1, or = 1 indicates positive, negative, or neutral evolution, respectively. Furthermore, Tajima relative rate tests were performed to determine the equality of the evolutionary rate between *GhAPX* paralogues and orthologues.

### RNA extraction and qRT-PCR

Total RNA was extracted from different cotton tissues by a modified hot borate method as described (Shi et al. 2006). Five micrograms of total RNA for each tissue were used to synthesize first-strand cDNA using SuperScript^®^ III first-strand synthesis system for RT-PCR (Invitrogen, Carlsbad, CA, USA). Reverse transcript PCR (RT-PCR) and quantitative real-time PCR (qRT-PCR) were performed using the SYBR green real-time PCR master mixes (Appliedbiosystems, Foster, CA, USA) with specific primers provided in Table S1. The 5′- and 3′-UTR of *GhAPXs* were obtained by genome-referenced expressed sequence tags (ESTs) assembly as described to facilitate the gene-specific primer design (Jin et al. 2013). The *UBQ* gene was used as internal control to adjust the amount of template cDNA for quantitative analysis (Jin et al. 2016). The relative expression level of each *APX* gene was used to generate a heat map using MultiExperiment viewer (MeV, version 4.9) software.

### *Cis-*regulatory elements analysis

The promoters of *GhAPXs* were downloaded in local database and the Plant CARE database (http://bioinformatics.psb.ugent.be/webtools/plantcare/html/) was used to analyze the *cis*-regulatory element of *GhAPX*s promoter (Lescot et al. 2002).

### Determination of APX enzyme activity and H_2_O_2_ content

Different fiber tissues (5d, 10d, 15d, 20d, 25d, and 30d) were used to measure the APX enzyme activity and H_2_O_2_ content as described (Li et al. 2007).

## Results

### Identification of the *APX* gene family in *G. hirsutum*

Several reports indicated that *GhAPX1* plays important roles during cotton fiber initiation and elongation stages (Fig. S1) (Shi et al. 2006; Li et al. 2007; Yang et al. 2008; Zheng et al. 2014; Guo et al. 2016). To further understand the functional of *APX* gene family, a genome-wide investigation of *GhAPXs* was performed. A total of 26 non-redundant *GhAPX* genes (Table S2) were identified by searching the cotton genome database, and were renamed from *GhAPX1A* to *GhAPX13A* and from *GhAPX1D* to *GhAPX13D* according to their order in chromosomes of the A and D sub-genomes except for previously reported *GhAPX1A* and *GhAPX1D*. The detailed information of these genes were listed in Table 1, including chromosome location, ORF length, protein length, molecular weight, and theoretical isoelectric point.

**Table 1:** List of *APX* genes in *G. hirsutum*

Gene name	Gene locus	Chr^a^	ORF (bp)	Predicted protein		
				Length (aa)	MW (kD)	*p*I
*GhAPX1A*	Gh_A05G0863	A-Chr5	753	250	27.58	5.93
*GhAPX2A*	Gh_A02G1648	A-Chr2	909	302	33.98	8.81
*GhAPX3A*	Gh_A03G1812	A-Chr3	867	288	31.94	6.67
*GhAPX4A*	Gh_A04G0652	A-Chr4	552	183	20.54	7.69
*GhAPX5A*	Gh_A01G1388	A-Chr1	867	288	31.86	5.55
*GhAPX6A*	Gh_A05G3726	A-Chr5 scaffold1211	1182	393	42.73	9.04
*GhAPX7A*	Gh_A06G0270	A-Chr6	1068	355	38.83	8.13
*GhAPX8A*	Gh_A06G0383	A-Chr6	1167	388	42.25	7.13
*GhAPX9A*	Gh_A06G2046	A-Chr6 scaffold1353	960	319	34.41	8.78
*GhAPX10A*	Gh_A08G1744	A-Chr8	753	250	27.53	5.51
*GhAPX11A*	Gh_A08G1745	A-Chr8	726	241	26.72	7.18
*GhAPX12A*	Gh_A08G1746	A-Chr8	867	288	32.07	6.42
*GhAPX13A*	Gh_A13G2003	A-Chr13	762	253	28.00	5.30
*GhAPX5D*	Gh_D01G1632	D-Chr1	867	288	31.78	5.61
*GhAPX2D*	Gh_D03G0074	D-Chr3	912	303	33.95	7.78
*GhAPX3D*	Gh_D02G2245	D-Chr2	867	288	31.96	6.67
*GhAPX4D*	Gh_D04G1116	D-Chr4	723	240	26.55	6.07
*GhAPX6D*	Gh_D05G2244	D-Chr5	1218	405	40.82	8.86
*GhAPX1D*	Gh_D05G3875	D-Chr5 scaffold4074	753	250	27.56	5.73
*GhAPX7D*	Gh_D06G0293	D-Chr6	1065	354	38.71	6.66
*GhAPX8D*	Gh_D06G0413	D-Chr6	1353	450	49.68	6.78
*GhAPX9D*	Gh_D06G1049	D-Chr6	1008	335	36.10	9.41
*GhAPX10D*	Gh_D08G2093	D-Chr8	741	246	27.12	5.72
*GhAPX11D*	Gh_D08G2094	D-Chr8	738	245	27.02	5.67
*GhAPX12D*	Gh_D08G2095	D-Chr8	879	292	32.56	6.46
*GhAPX13D*	Gh_D13G2402	D-Chr13	750	249	27.31	5.29

^a^Chromosome number in which the gene anchors. *A* sub-genome A, *D* sub-genome D, *ORF* open reading frame length, *aa* amino acid, *MW* molecular weight, *pI* theoretical isoelectric point

**Fig. 1:**
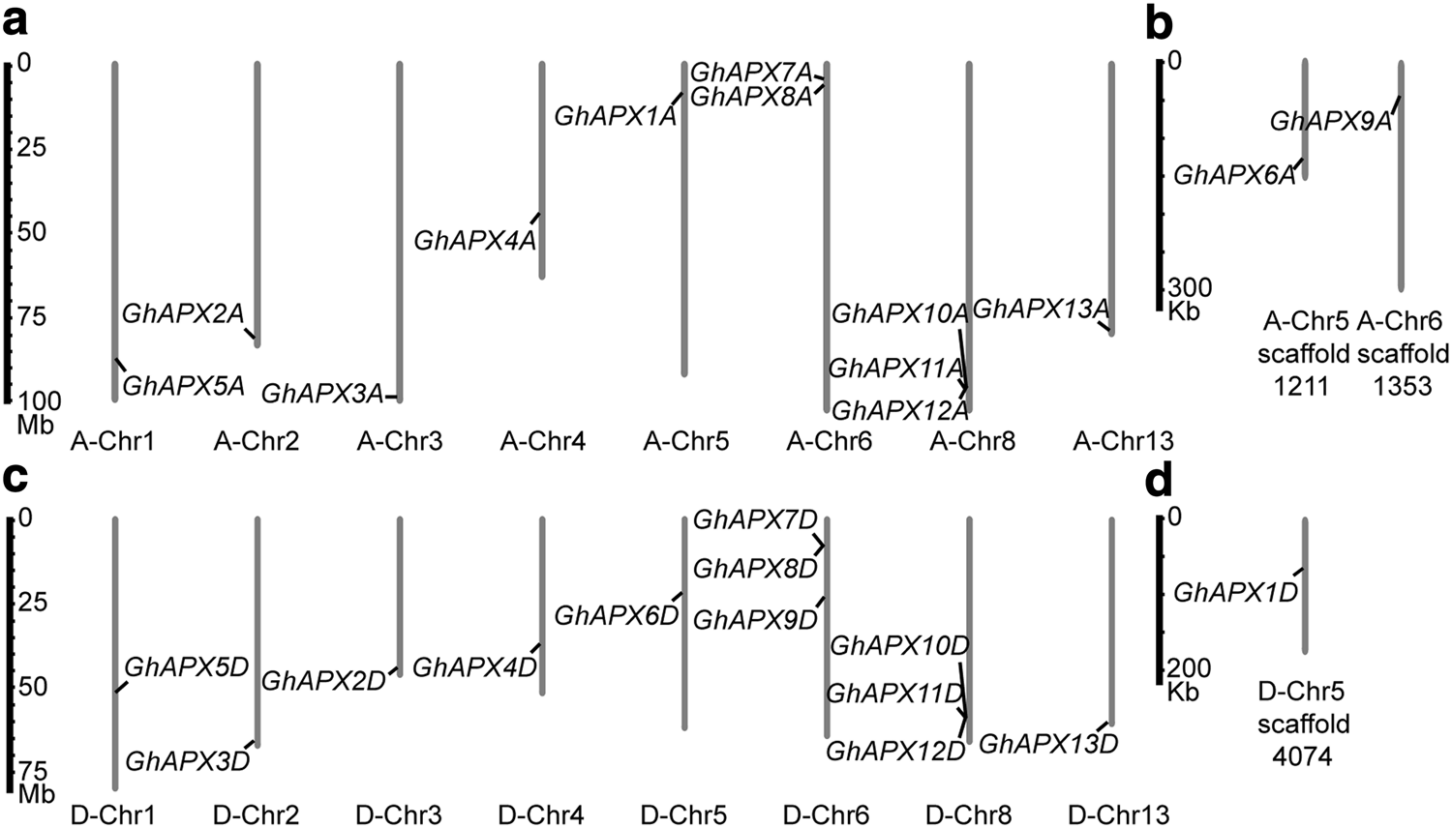
Chromosome distribution of *GhAPX* gene family. The 26 *Gh*APX genes were mapped to the chromosomes of A sub-genome (**a**) and un-assembled scaffolds (**b**), as well as that of D sub-genome (**c**) and un-assembled scaffold (**d**). Genes were referred as *GhAPX1A–13A* and *GhAPX1D–13D*, according to their organization order on the chromosomes, except for the previously reported *GhAPX1A*/*D*. Different scales were used for chromosomes and un-assembled scaffolds. Note that the chromosome information of scaffolds was known, although they could not be assembled to the corresponding chromosomes

Chromosome distribution analysis showed that *GhAPX1A–GhAPX5A, GhAPX7A, GhAPX8A*, and *GhAPX10A*–*13A* were anchored in eight chromosomes of A sub-genome, while *GhAPX6A* and *GhAPX9A* were anchored in two un-assembled scaffolds: A-Chr5 scaffold1211 and A-Chr6 scaffold1353, respectively (Fig. 1a, b). For D sub-genome, *GhAPX2D*–*GhAPX13D* were anchored in eight chromosomes, and *GhAPX1D* was anchored in D-Chr5 scaffold4074 (Fig. 1c, d). The 26 GhAPXs were predicted to be located in different apparatus including 18 (9 ortholog pairs) in cytoplasm, four in periplasm, and four in outer membrane (Table S3).

### Phylogenetic and intron–exon distribution analyses of *GhAPX* gene family

**Fig. 2:**
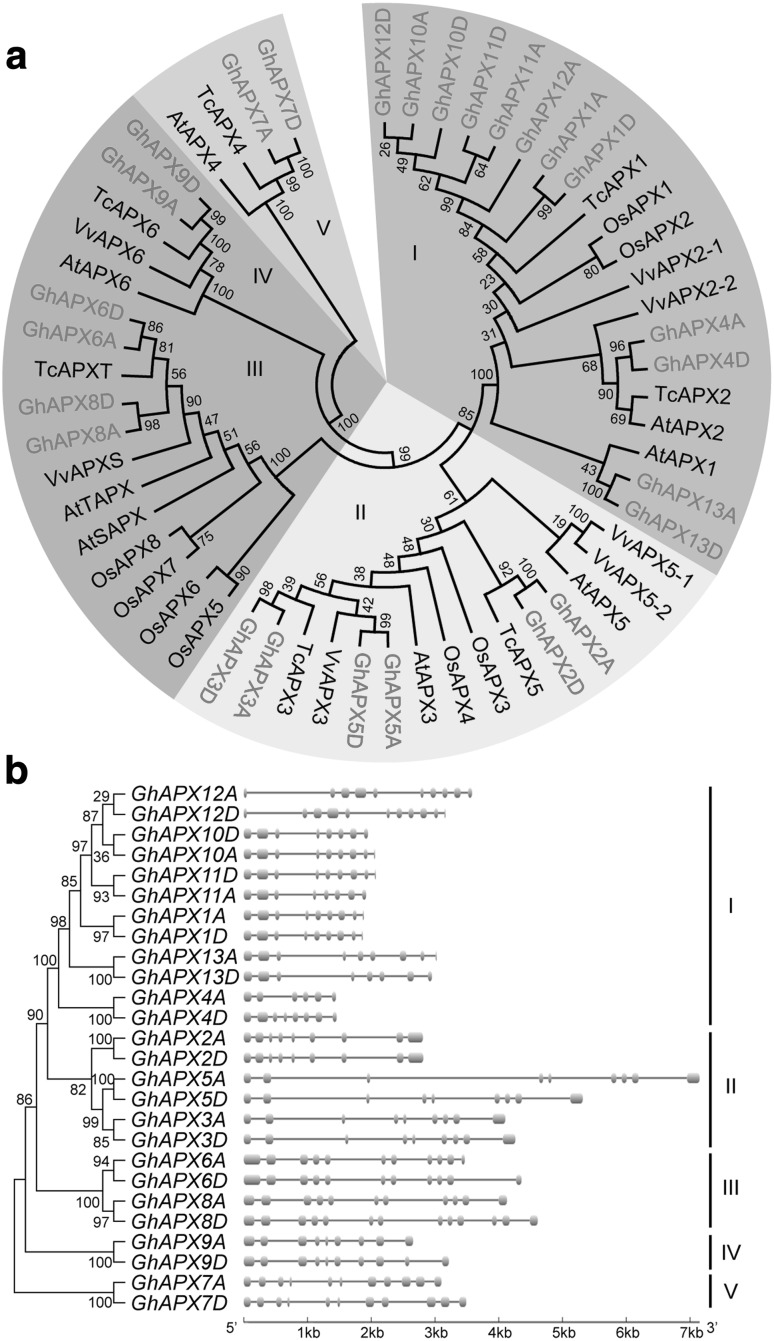
Phylogenetic and intron–exon structure analyses of *APX* family genes. **a** Protein sequences of APX gene families from *Arabidopsis thaliana* (AtAPX), *G. hirsutum* (GhAPX), *Oryza sativa* (OsAPX), *Theobroma cacao* (TcAPX), and *Vitis vinifera* (VvAPX) were used to construct an NJ phylogenetic tree. Bootstrap was set to 1000 replicates. Five clades were named as sub-family I to V, shadowed with different colours. **b** Intron–exon organization structure analysis of the 26 *GhAPX* genes was shown. Boxes and lines represented the exons and introns, and the genomic length was indicated at the bottom. (Color figure online)

To in-depth understand the evolutionary and phylogenetic relationships of *GhAPXs*, a neighbor-joining (NJ) phylogenetic tree was constructed using protein sequences of APXs from *Arabidopsis thaliana, G. hirsutum, O. sativa, Vitis vinifera, and T. cacao* (Fig. 2a). The 26 GhAPXs could be classified into five well-supported clades labeled with different colours. According to *A. thaliana* APXs (Panchuk et al. 2005), clade I and clade II consist of cytoplasmic APXs, Clade III contains all known chloroplast APXs, and Clade IV and V include peroxisomal APXs. These phylogenetic data demonstrated similar conclusions to a previously published work (Guo et al. 2016).

Gene structures of all the 26 *GhAPXs* were investigated to further validate the evolution and phylogenetic relationships of *GhAPX* family members. The *GhAPX* genes belonged to the same clade in phylogenetic tree shared similar intron–exon organization structures (Fig. 2b).

### Syntenic and evolutionary analyses of *GhAPX* gene family

**Fig. 3:**
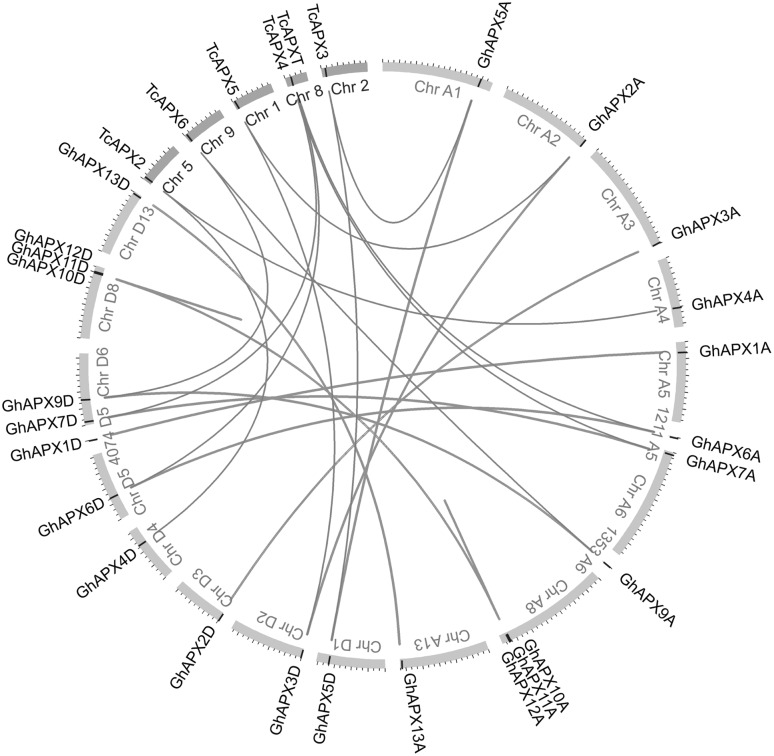
Tandem duplication and segmental duplication of *GhAPXs* and syntenic analysis between *G. hirsutum* and *T. cacao APXs*. Chromosomes and scaffolds from cotton (Gh) and cacao (Tc) are shown in yellow and blue segments, respectively. The positions of the APX genes are marked by black lines in the circus. Duplicated *GhAPXs* are linked by red lines and syntenic relationships between cotton and cacao are linked by purple lines. (Color figure online)

To investigate the expansion of the *APX* gene family, syntenic analysis of *G. hirsutum* and *T. cacao APXs* was performed using Circos software. Two tandem duplication events were detected in both A- and D-sub-genome (*GhAPX10A*/*11A*/*12A* and *GhAPX10D*/*11D*/*12D*); however, no segmental duplication events were determined because of the high conservation of *GhAPX*s between A- and D-sub-genome (Fig. 3).

Evolutionary selection patterns between paralogue and/or orthologue gene pairs can be estimated by the *Ka*/*Ks* ratio (Yadav et al. 2015). A *Ka*/*Ks* ratio > 1 indicates a positive selection, a *Ka*/*Ks* ratio < 1 indicates a purifying selection, and a *Ka*/*Ks* ratio = 1 indicates a neutral selection. The *Ka*/*Ks* ratios of the duplicated *GhAPXs* indicated that they all were subjected to purifying selection (Table 2). In addition, Tajima relative rate were calculated to determine whether the *GhAPX* duplicates evolved at an accelerated rate following the duplication events. Notably, statistically significant increase in evolutionary rate occurred between the *GhAPX10A*/*11A*/*12A* duplicated paralogues, while non-significant evolutionary rate occurred between *GhAPX10D*/*11D*/*12D* (Table 3), indicating a potential functional divergence of these duplicated paralogues.

**Table 2:** *Ka*/*Ks* ratios for duplicate APX genes in *G. hirsutum*

Paralogous genes	*Ka*	*Ks*	*Ka*/*Ks*	Selective pressure
*GhAPX1A*/*GhAPX1D*	0.0065	0.0214	0.3037	Purity selection
*GhAPX2A*/*GhAPX3D*	0.0166	0.0317	0.5237	Purity selection
*GhAPX3A*/*GhAPX2D*	0.0098	0.0326	0.3006	Purity selection
*GhAPX5A*/*GhAPX5D*	0.0131	0.0436	0.3005	Purity selection
*GhAPX6A*/*GhAPX6D*	0.0064	0.0472	0.1356	Purity selection
*GhAPX7A*/*GhAPX7D*	0.0131	0.0329	0.3982	Purity selection
*GhAPX9A*/*GhAPX9D*	0.0861	0.1498	0.5748	Purity selection
*GhAPX10A*/*GhAPX10D*	0.0032	0.0220	0.1456	Purity selection
*GhAPX12A*/*GhAPX12D*	0.0065	0.0333	0.1952	Purity selection
*GhAPX13A*/*GhAPX13D*	0.0263	0.0444	0.5923	Purity selection
*GhAPX10A*/*GhAPX11A*	0.0295	0.0569	0.5185	Purity selection
*GhAPX10D*/*GhAPX11D*	0.0229	0.0448	0.5112	Purity selection
*GhAPX10A*/*GhAPX12A*	0.0065	0.0221	0.2941	Purity selection
*GhAPX10D*/*GhAPX12D*	0.0032	0.0109	0.2935	Purity selection
*GhAPX11A*/*GhAPX12A*	0.0295	0.0571	0.5167	Purity selection
*GhAPX11D*/*GhAPX12D*	0.0169	0.0564	0.2996	Purity selection

*Ka* nonsynonyomous subsititution rate, *Ks* synonyomous subsititution rate

**Table 3:** Tajima relative rate tests of APX gene pairs in cotton

Testing group	Mt^a^	M1^b^	M2^c^	*X* ^2^	*P* ^d^
*GhAPX1A*/*GhAPX1D* with *TcAPX1*	235	1	0	1.00	0.31731
*GhAPX2A*/*GhAPX3D* with *TcAPX5*	230	2	2	0.00	1.00000
*GhAPX3A*/*GhAPX2D* with *TcAPX3*	269	1	1	0.00	1.00000
*GhAPX5A*/*GhAPX5D* with *TcAPX3*	248	6	5	0.09	0.76302
*GhAPX6A*/*GhAPX6D* with *TcAPXT*	308	7	6	0.08	0.78151
*GhAPX7A*/*GhAPX7D* with *TcAPX4*	297	4	2	0.67	0.43858
*GhAPX9A*/*GhAPX9D* with *TcAPX6*	252	5	4	0.11	0.73888
*GhAPX10A*/*GhAPX10D* with *TcAPX1*	226	1	1	0.00	1.00000
*GhAPX12A*/*GhAPX12D* with *TcAPX1*	224	2	2	0.00	1.00000
*GhAPX10A*/*GhAPX11A* with *TcAPX1*	198	2	23	17.64	**0.00003**
*GhAPX10D*/*GhAPX11D* with *TcAPX1*	218	1	4	1.80	0.17971
*GhAPX10A*/*GhAPX12A* with *TcAPX1*	225	2	1	0.33	0.56370
*GhAPX10D*/*GhAPX12D* with *TcAPX1*	225	1	2	0.33	0.56370
*GhAPX11A*/*GhAPX12A* with *TcAPX1*	197	23	3	15.38	**0.00009**
*GhAPX11D*/*GhAPX12D* with *TcAPX1*	220	5	3	0.50	0.47950
*GhAPX13A*/*GhAPX13D* with *TcAPX1*	183	5	11	2.25	0.13361

The Tajima relative rate test was used to examine the equality of evolutionary rate between cotton paralogs

^a^Mt is the sum of the identical sites in all three sequences tested

^b^M1 is the number of unique differences in the first paralog

^c^M2 is the number of unique differences in the second paralog

^d^If *P* < 0.05 were indicated as significant variable and they are in bold, the test rejects the equal substitution rates between the two duplicates and infers that one of the two duplicates has an accelerated evolutionary rate

### Conserved motif analysis of GhAPX family

Multiple sequence alignment showed that all GhAPXs had three conserved domains and variable N-terminus (Fig. S2). Domain I contains two active sites, Domain II has the most conserved 12 amino acids sequences that predicted as Heme-binding site, and Domain III has three proximal cation-binding sites. These data provide possibility that GhAPXs may function in different organelles using ascorbate as substrate to detoxify H_2_O_2_.

**Fig. 4:**
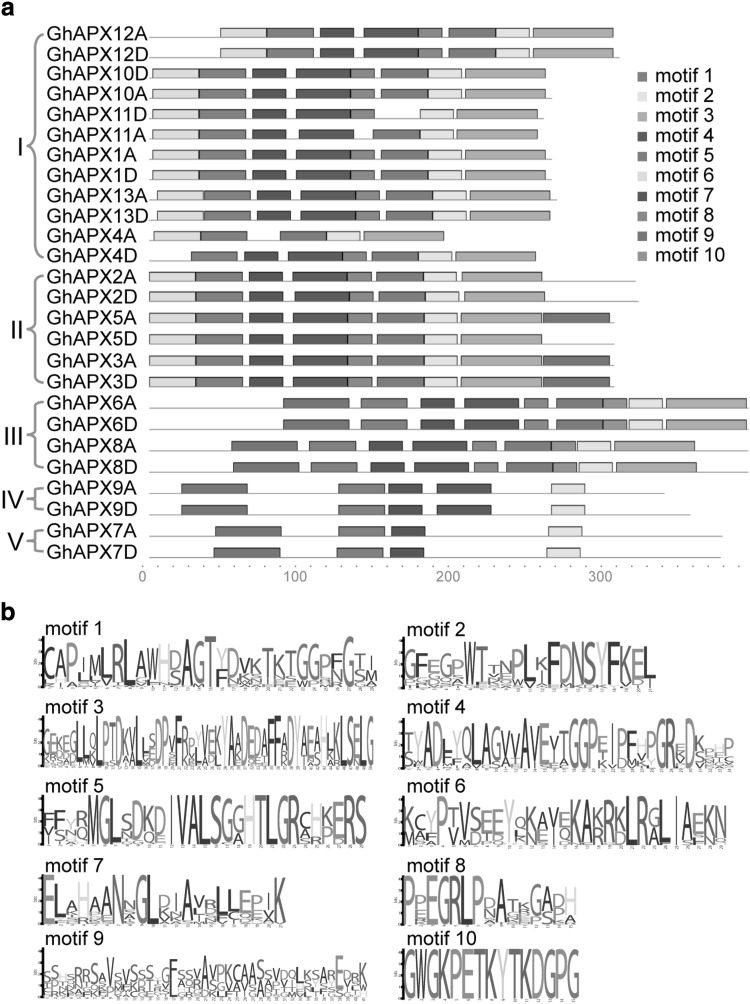
Motif analysis of GhAPXs. Conserved motifs of GhAPX protein sequences were analyzed (**a**). Ten different motifs were recognized and indicated with different colours. The organization order of motifs for each member of GhAPXs was highlighted. The conservation of the sequences for each conserved domain was also presented (**b**). (Color figure online)

Conserved motifs in GhAPXs were searched by MEME program to obtain more insights into the diversity of motif compositions and evolutionary relationships, and a total of ten conserved motifs were discovered. The APXs belong to the same clades that share very similar motif composition and order (Fig. 4a). Motif 1, 2, and 7 are existed in all GhAPXs, indicating that they are conserved sections of GhAPXs. Most of cytoplasmic GhAPXs have motifs 1–8, except for orthologs GhAPX4A and GhAPX4D. Chloroplast GhAPXs have all motifs but not motif 6, while the peroxisomal APXs have the least conserved motifs 1, 2, 7, and 9. Motif 10 is only distributed in the chloroplast-located APXs, with a most conserved amino acid sequence of GWGKPETKYTKDGPG (Fig. 4a, b). Motif 9 is observed in the C-terminal of cytoplasmic GhAPX3A/D and GhAPX5A, while in the N-terminal of the peroxisome- and chloroplast-located GhAPXs.

### Tissue- and development-specific expression profiling of *GhAPX* genes

**Fig. 5:**
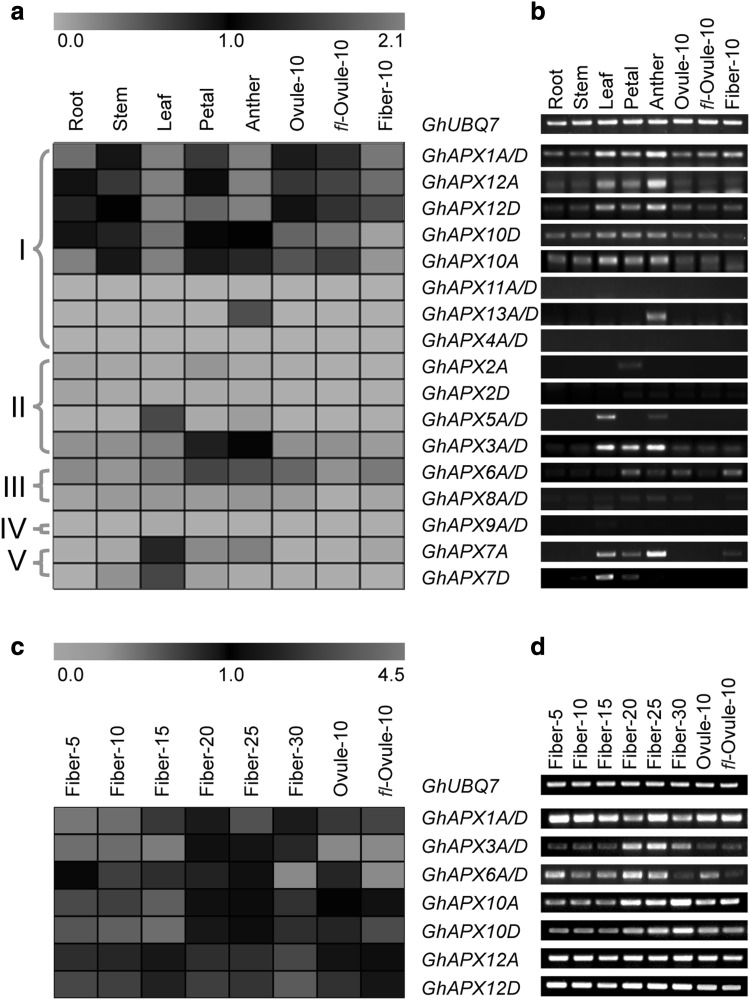
Expression profiling of *GhAPX* genes in different cotton tissues and different development stage of cotton fibers. **a** Heat map of the qRT-PCR data for the 26 *GhAPXs* in eight different cotton tissues. Orthologs with very high identities that could not be distinguished by gene-specific primers were examined together use the same primers and indicated by slashes. **b** Semi-quantitative RT-PCR results of *GhAPX* genes corresponding to that of in **a. c** Heat map of the qRT-PCR data for ten fiber-preferentially accumulated *GhAPXs* in eight development stages of cotton fibers. Orthologs *GhAPX1A*/*D, GhAPX3A*/*D*, and *GhAPX6A*/*D* were detected use the same primers, respectively. **d** Semi-quantitative RT-PCR results of *GhAPX* genes corresponding to that of in **c**. Relative expression levels were normalized by the internal control *UBQ* gene

To understand the expression and function diversity of the 26 *GhAPX* genes, the tissue- and development-specific expression profiles of *GhAPXs* were performed using qRT-PCR. Relative expression levels of the 26 *GhAPX* genes in eight different tissues were demonstrated to construct a heat map (Fig. 5a). All expression level data were normalized using cotton *UBQ* as internal control, and relative expression level over 0.05-fold to *UBQ* was considered to be detected. Members of cytoplasmic *GhAPX* sub-family I were universal expressed, in which *GhAPX1A*/*D, GhAPX10A, GhAPX10D, GhAPX12A*, and *GhAPX12D* were the predominantly expressed *GhAPXs*, with high levels in leaf, petal, and anther. Members of cytoplasmic *GhAPX* sub-family II were expressed in much less tissues: *GhAPX5A*/*D* was expressed only in leaf, and *GhAPX3A*/*D* was expressed in leaf, petal, and anther, indicating the functional diversity of the two clusters of *GhAPXs*. However, none of chloroplast *GhAPXs* were detected, except for *GhAPX6A*/*D*. For peroxisomal *GhAPX* sub-family IV and V, *GhAPX7A* was expressed in leaf and anther, while *GhAPX7D* expressed only in leaf. The semi-quantitative RT-PCR results were also showed with visible confirmation (Fig. 5b).

Interestingly, ten *GhAPX* genes were detected in 10-day fibers (Fiber-10), and selected to further examine the expression patterns during cotton fiber development. Fibers of eight developmental stages were used for qRT- and RT-PCR analyses, including 5-day (Fiber-5), 10-day (Fiber-10), 15-day (Fiber-15), 20-day (Fiber-20), 25-day (Fiber-25), 30-day (Fiber-30), 10-day WT ovule (Ovule-10), and 10-day *fl* ovule (*fl*-Ovule-10). The results showed that these ten *GhAPX* genes displayed three distinct expression patterns according to different temporal expression feature during fiber development stages. *GhAPX1A*/*D* had a predominant steady expression level from 5 days to 25 days of fiber elongation and secondary cell wall biosynthesis. Notably, *GhAPX6A*/*D* and *GhAPX3A*/*D* were mainly expressed at the late fiber development stage of secondary cell wall biosynthesis (Fig. 5c, d). Meanwhile, *GhAPX10A, GhAPX10D, GhAPX12A*, and *GhAPX12D* had the highest expression level at 30 days, the cell apoptosis stage of fiber development. The results of the 26 *GhAPX* gene expression patterns provide their probable multiple functions in cotton plant development, particularly the potential diverse role in controlling H_2_O_2_ concentration during different fiber development stages.

### Determination of APX activity and H_2_O_2_ content during fiber development

**Fig. 6:**
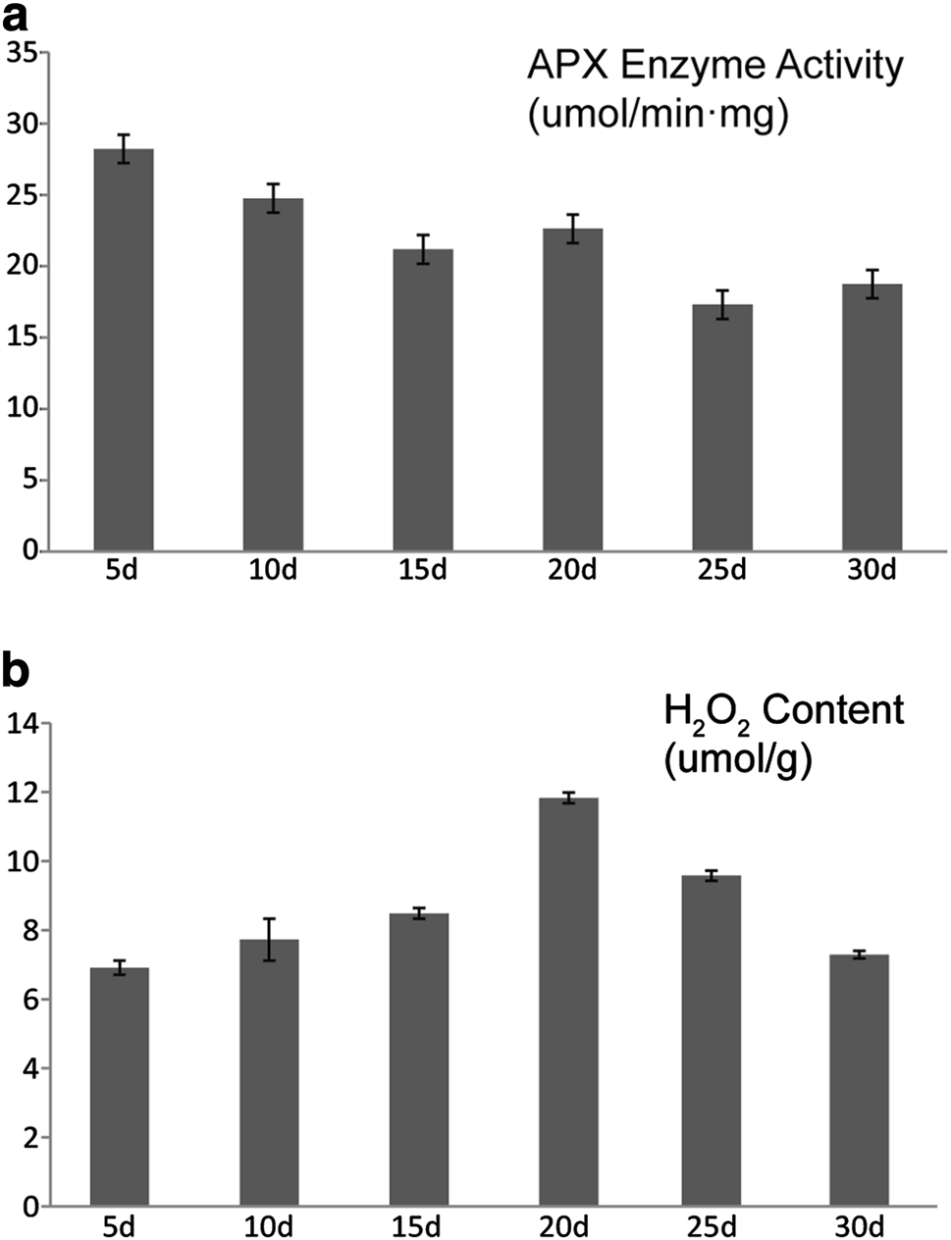
Dynamic changes of the APX enzyme activity and H_2_O_2_ content in different stages of cotton fibers. **a** Dynamic changes of APX enzyme activity in different stages of cotton fibers (from 5 to 30 dpa). **b** Dynamic changes of H_2_O_2_ content in different stages of cotton fibers (from 5 to 30 dpa)

To understand the relationship between APX expression and H_2_O_22_homeostasis during fiber development, different tissues of 5d, 10d, 15d, 20d, 25d, and 30d fibers were collected to measure the APX activity and H_2_O_2_ content. The level of APX activity reached the peak value in 5d fibers, and maintained a steady high expression with a tendency of decline at fast fiber elongation stages (5–15 dpa), following a slight increase at 20 dpa, which is matched well to the gene expression level of *GhAPXs*, indicating that there may be diverse *GhAPXs* functioning in different developmental stages (Fig. 6a). Meanwhile, H_2_O_2_ content demonstrated an ascending trend at the fast fiber elongation stages (5–15 dpa) with highest concentration in 20d fibers, and then decrease gradually at the secondary cell wall synthesis stages (20–30 dpa). The results imply the possibility that some *GhAPXs* accumulated in the secondary cell wall biosynthesis and maturation stages of fiber development may involve in H_2_O_2_ scavenging (Fig. 6b).

### Analyses of *cis-*regulatory element and gene expression profiling of *GhAPXs* in response to stimulations of H_2_O_2_ and phytohormone

**Fig. 7:**
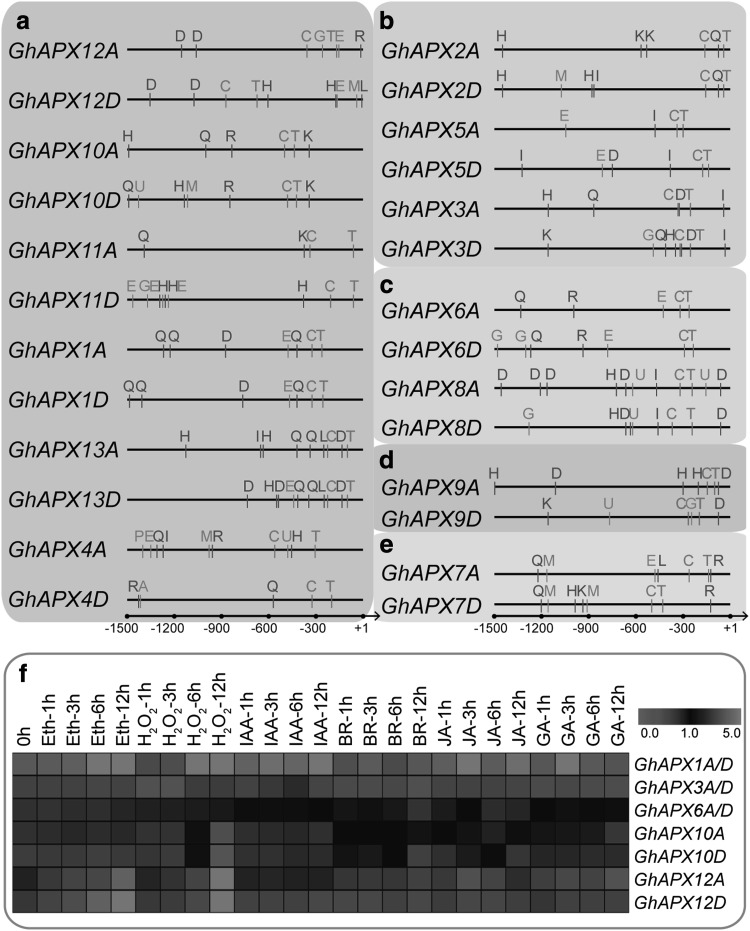
*Cis*-regulatory elements analysis and expression profiling of *GhAPXs* response to plant hormone (**a**–**e**). The putative *cis*-regulatory elements of *GhAPXs* were labeled with capital letters in the figure according to their relative position. The green letters stand for stress response *cis*-elements, the blue letters stand for plant hormone response *cis*-elements, and the red letters stand for transcription initiation *cis*-elements. More details about *cis*-regulatory elements are shown in S4 Table. **f** Heat map of the qRT-PCR data for ten fiber-preferentially accumulated *GhAPXs* in 10-day fibers treated with different plant hormones at different hours. Orthologs *GhAPX1A*/*D, GhAPX3A*/*D*, and *GhAPX6A*/*D* were detected using the same primers, respectively. Relative expression levels were normalized by the internal control *UBQ* gene. (Color figure online)

To further investigate the regulatory mechanism of the *GhAPX* gene family members, especially the duplicated paralogues, the *cis*-elements were scanned in the promoter regions of *GhAPXs* (Fig. 7a–e). A 1500-bp sequence upstream of the translational start site was considered as a putative promoter region, and thus was used to analyze the distribution of *cis*-regulatory elements. The *cis*-elements were characterized and indicated with capital letters labeled by different colours, including two core *cis*-elements, nine stress response elements, and six phytohormone response elements which were characterized (Fig. 7, Table S4), which implies that the *GhAPXs* gene expression is under control of stimulation responsiveness of stress and phytohormone. Notably, in the process of cotton plant growth and development, similar *cis*-element distribution pattern was found in the promoter regions of the duplicated paralogues of common or higher expressing *GhAPX* genes, while the duplicated paralogues of lower or non-expressing *GhAPX* genes displayed different distributions with non-regular constitutions.

In view of H_2_O_2_ and phytohormone important functions in cotton fiber development, PKc enzyme activity is positive related to the H_2_O_2_ content and negatively correlated with fast fiber elongation in cotton. Phytohormones such as auxins, ethylene, and brassinosteroids are involved in regulation of fiber development (Shi et al. 2006; Pang et al. 2010; Chen and Guan 2011; Zhang et al. 2011, 2016). The expression patterns of *GhAPXs* in cotton fibers under oxidative stress and phytohormone stimulation were determined by treating 10-day WT fibers by H_2_O_2_, ETH, IAA, BR, JA and GA for 1, 3, 6, and 12 h. The results indicated that *GhAPX1A*/*D, GhAPX6A*/*D, GhAPX12A, GhAPX12D* were positively respond to ethylene stimulation. *GhAPX6A*/*D, GhAPX10A*, and *GhAPX12A* displayed induced expression after GA treatment, while *GhAPX10A, GhAPX10D*, and *GhAPX12A* were significantly increased after JA stimulation. No changes were detected after treatments of IAA and BR. Remarkably, all the fiber-expressed *GhAPX* members except for *GhAPX3A*/*D* illustrated significant induced expression after H_2_O_2_ treatment (Fig. 7f). These results suggested that *GhAPX* genes may perform multiple functions in the process of H_2_O_2_ and phytohormone regulated cotton fiber development. In addition, there exists the appearance that duplicated paralogues displayed different responsive characteristics, implying their functional and regulatory diversity (Fig. 7f).

## Discussion

DNA sequencing data of the cotton genome provide us valuable information of gene family in *Gossypium* to further understand gene function and regulation mechanism (Yao et al. 2012). Ascorbate peroxidase is known as the key enzyme detoxifying H_2_O_2_ and performs vital roles in plant growth and development and stress responsiveness (Fryer et al. 2003; Davletova et al. 2005). Whereas the diverse functions of *GhAPX* members remain unclear, especially in cotton fiber development. Thus, to comprehensively understand GhAPXs’ various roles and the regulatory mechanism, based on our previous study about *GhAPX1*, here, a complete overview of this *GhAPX* family in *G. hirsutum* is presented, as well as the expression profiling characteristics. Totally, 26 *APX* genes were identified according to the complete genome of *G. hirsutum*, locating onto 8 chromosomes of A- or D-sub-genome (Fig. 1; Table 1), while only 8, 8, 7, and 7 *APX* genes were characterized in *Arabidoisis, O. sativa, T. cacao*, and *V. vinifera*, respectively (Panchuk et al. 2005; Teixeira et al. 2006).

Phylogenetic analysis of the reported 74 APX members in different plant species showed that the APXs can be classified into four clades with different putative subcellular locations (Teixeira et al. 2004). However, the 26 cotton GhAPXs reported here were divided into five sub-families with putative different subcellular distributions according to the orthologous *APX* genes from *A. thaliana* (Fig. 2a). Intron–exon structure analysis presented high consistence with phylogenetic classification (Fig. 2b). Different APX sub-families displayed lower identities, indicating significant original and functional diversity of *GhAPX* gene family.

The alignment of 74 reported plant APX sequences revealed two signatures in plant chloroplast isoforms including 7 residues next to the active site (K-[ND]-I-[ETK]-E-W-P), and 16 residues near heme-binding site (E-T-K-Y-T-[KE]-[DNTE]-G-PG-[ANEK]-[PA]-G-G-Q-S), respectively. Phylogenetic analysis among different species showed that these 74 APXs were classified into 4 clades with different subcellular locations (Teixeira et al. 2004). We found that all APX proteins contain three conserved domains (Fig. S2). Study of *APX* gene family in *O. sativa* illustrated that a sequence of gene duplications led to the current diversity of isoforms (Teixeira et al. 2004), suggesting that these unique motifs may be responsible for diverse functions in different isoforms. The specific exon I and II of chloroplast *APX* gene Sl*Apx7* and Sl*Apx6* encoded for the organellar targeting sequences of the proteins (Najami et al. 2008). Under these circumstances, we deduce that the particular conserved motifs in chloroplast and cytoplasmic GhAPXs, as well as different intron–exon structures (Fig. 2 and Table S3) may have their specific possible action in targeting the organelles.

Gene expression patterns are usually closely related to their functions, and analyses of differential expression profiles can provide important information with gene families. (Guo et al. 2008). Eight APX members containing three cytosolic, two chloroplastic, and three microsomal isoforms were characterized in *A. thaliana*, in which *APX1* and *APX3* were appeared to be high expressed, while *APX2* and *APX5* were low expressed between different age leaves (Panchuk et al. 2005). KO-*APX1* experiment in *A. thaliana* showed that cytosolic APX1 plays an important role in protecting chloroplast from H_2_O_2_ damage, and stromal/mitochondrial APX can be the first chloroplast line to defend against the diffusion of H_2_O_2_ from cytosol into the chloroplast (Davletova et al. 2005). In *Solanum lycopersicum*, dominant expressions of *SlAPX6* in leaves and *SlAPX7* in stems were observed (Najami et al. 2008). Members of *GhAPX* family were characterized with different expression profiles in root, leaf, anther, and fiber, ten *GhAPX* genes demonstrated fiber-specific expressions with distinct patterns according to their abundant accumulation in different stages of fiber development (Fig. 5). It has been demonstrated that *GhAPX1* is highly up-regulated during fiber fast elongating stages (Li et al. 2007; Yang et al. 2008; Zheng et al. 2014; Guo et al. 2016), which is consistent with the current result that *GhAPX1A*/*D* are mainly expressed in the elongation and secondary cell wall biosynthesis stages of fiber development. Interestingly, in the secondary cell wall biosynthesis and maturation stages of fiber development, four genes of *GhAPX10A*/*D* and *GhAPX12A*/*D* were mostly enriched; meanwhile, H_2_O_2_ content indicated a tendency of decline (Fig. 6), indicating potential possibility that the four *GhAPXs* may be major members controlling intracellular H_2_O_2_ levels in the maturation stages of fiber development.

H_2_O_2_ and phytohormone are key factors in regulating fiber development (Li et al. 2007; Triplett et al. 2007). Many genes have been investigated that perform essential functions in fiber development through responding to phytohormone, cotton *CesA* were reported to increase fiber number per seed after auxin and gibberellin treatments (Triplett et al. 2007), gibberellin could induce significant expression of cotton *KCS* gene in cotton fibers (Xiao et al. 2016), and the transcription of the cotton *AOCs* was increased after JA treatment (Wang et al. 2015). Our previous work showed that *GhAPX1* is involved in the response to ethylene and H_2_O_2_ stimulations (Li et al. 2007). The current investigations of the expression profiles of the *APX* genes in response to phytohormone and H_2_O_2_ treatments indicated that fiber-expressed *GhAPXs* are expressed under control of ethylene, GA, and JA. Notably, fiber-expressed *GhAPXs* except for *GhAPX3A*/*D* were responded to H_2_O_2_ stimulation, suggesting that these *GhAPXs* may be participated in redox homeostasis. Distribution analysis of *cis*-elements of the promoter regions of *GhAPX* genes supplies potential regulation mechanism of APX responding to H_2_O_2_ and phytohormone (Fig. 7). In summary, through analyses of genome-wide survey and expression profiling of *GhAPX* gene family, we provided some new insights in controlling H_2_O_2_ homeostasis during fiber development, that is decided by the ten fiber-preferentially accumulated *GhAPXs*.

In conclusion, we performed thoroughly investigation of upland cotton *GhAPX* gene family. The evolutionary analyses suggested a significant increase in evolutionary rate between the A-sub-genome duplicated paralogue genes *GhAPX10A*/*11A*/*12A*, while non-significant evolutionary rate between *GhAPX10D*/*11D*/*12D*. Tissue- and development-specific expression profiling of *GhAPX* genes revealed that 10 members were expressed in cotton fiber and *GhAPX10A, GhAPX10D, GhAPX12A*, and *GhAPX12D* showed high expression levels in 30-day fiber, while *GhAPX1A*/*D, GhAPX3A*/*D*, and *GhAPX6A*/*D* showed relative low expression levels. Together with the APX enzyme activity and H_2_O_2_ content assay, we demonstrated that different *GhAPX* family members are responsible for redox homeostasis during different cotton fiber development stages.

## Electronic supplementary material

Below is the link to the electronic supplementary material.

**Previously reported APX1 gene/protein expression patterns in** ***G. hirsutum***. Expression patterns of *G. hirsutum* APX1 gene/protein in different cotton fiber development stages were deduced from five previous references using qRT-PCR (Li et al. 2007; Zheng et al. 2014), northern blot (Guo et al. 2016), microarray (Shi et al. 2006), and two-dimensional electrophoresis (Yang et al. 2006). The expression levels of GhAPX1 in 0-day ovule and 5-day fibers were set to 1, according to the authors (TIF 253 KB)

**Multiple sequence alignment of GhAPXs**. Multiple sequence alignment was performed using the protein sequences of the 26 GhAPXs. Conserved domains I, II, and III were underlined. The label “H” marked the heme-binding amino acids, “D” the distal cation-binding sites, “A” ascorbate binding amino acids, “C” the amino acids involved in formation of the catalytic site, “P” the proximal cation biding sites (TIF 1830 KB)

**Expression analysis of** ***GhAPX*** **genes using transcriptome and EST data**. (a) The EST frequencies for each *GhAPX* gene were determined by screening over 0.3 million EST sequences downloaded from GenBank EST database. Higher EST hits numbers were considered to represent higher expression levels. Note the significant frequency difference between orthologs *GhAPX11A* and *GhAPX11D*, which is consistent with the qRT-PCR data shown in Fig. 4. (b) Heat map profiling of 26 *GhAPX* genes using RNA-seq data downloaded from genome database of *G. hirsutum* TM-1. The reads per kilobases per millionreads (RPKM) values were used to estimate the expression level of each *GhAPX* in different cotton tissues (TIF 1275 KB)

Supplementary material 4 (XLSX 10 KB)

Supplementary material 5 (XLSX 19 KB)

Supplementary material 6 (XLSX 12 KB)

Supplementary material 7 (XLSX 10 KB)
