# Supplementary figures and images for: Genome-wide investigation and expression profiling of APX gene family in Gossypium hirsutum provide new insights in redox homeostasis maintenance during different fiber development stages
Source: Mol Genet Genomics. 2018 Jan 6;293(3):685–97. doi: 10.1007/s00438-017-1413-2 (PMC5948307; doi:10.1007/s00438-017-1413-2)

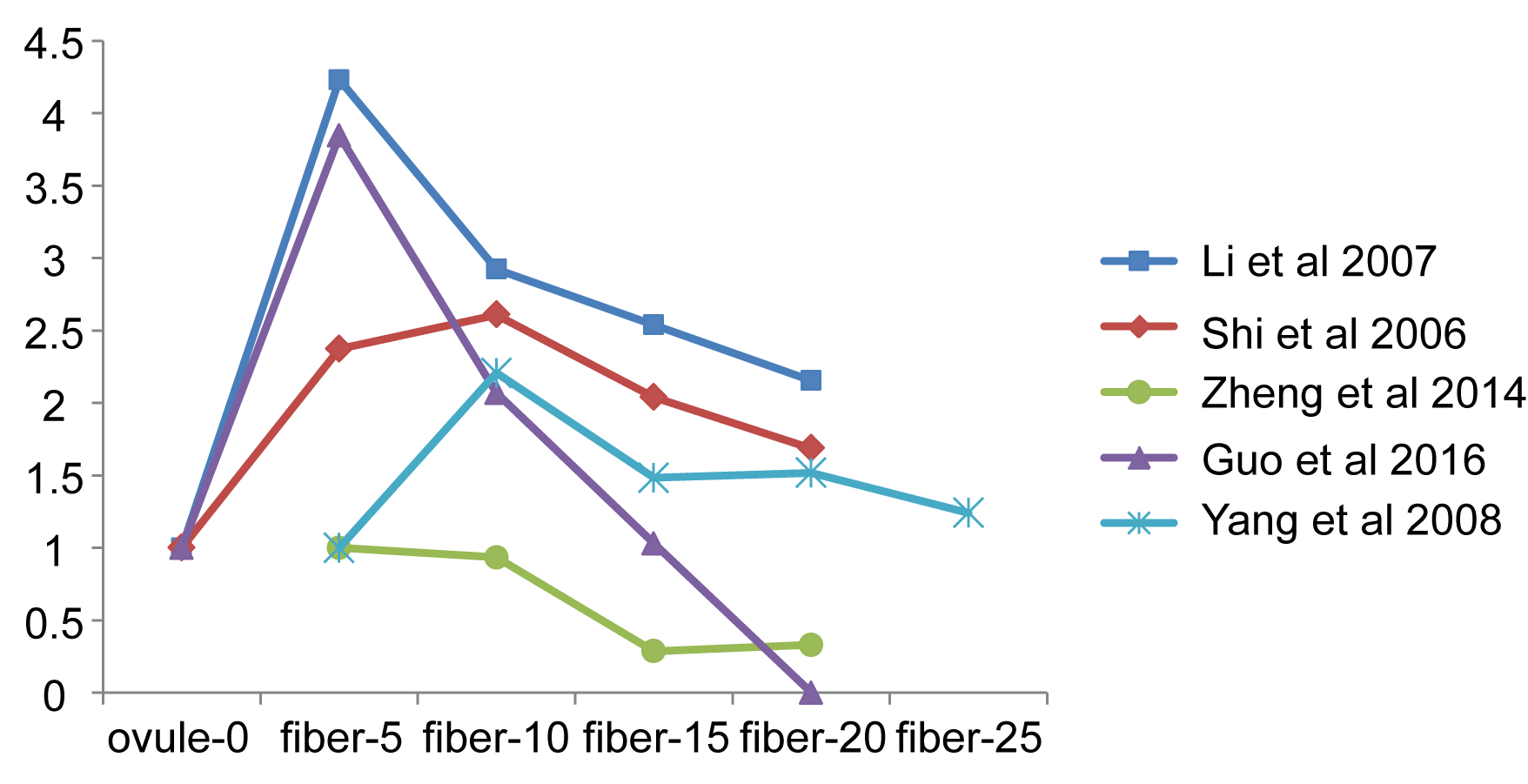

Supplement: Supplementary file 1 — Previously reported APX1 gene/protein expression patterns in G. hirsutum. Expression patterns of G. hirsutum APX1 gene/protein in different cotton fiber development stages were deduced from five previous references using qRT-PCR (Li et al. 2007; Zheng et al. 2014), northern blot (Guo et al. 2016), microarray (Shi et al. 2006), and two-dimensional electrophoresis (Yang et al. 2006). The expression levels of GhAPX1 in 0-day ovule and 5-day fibers were set to 1, according to the authors (TIF 253 KB) [file 438_2017_1413_MOESM1_ESM.tif]

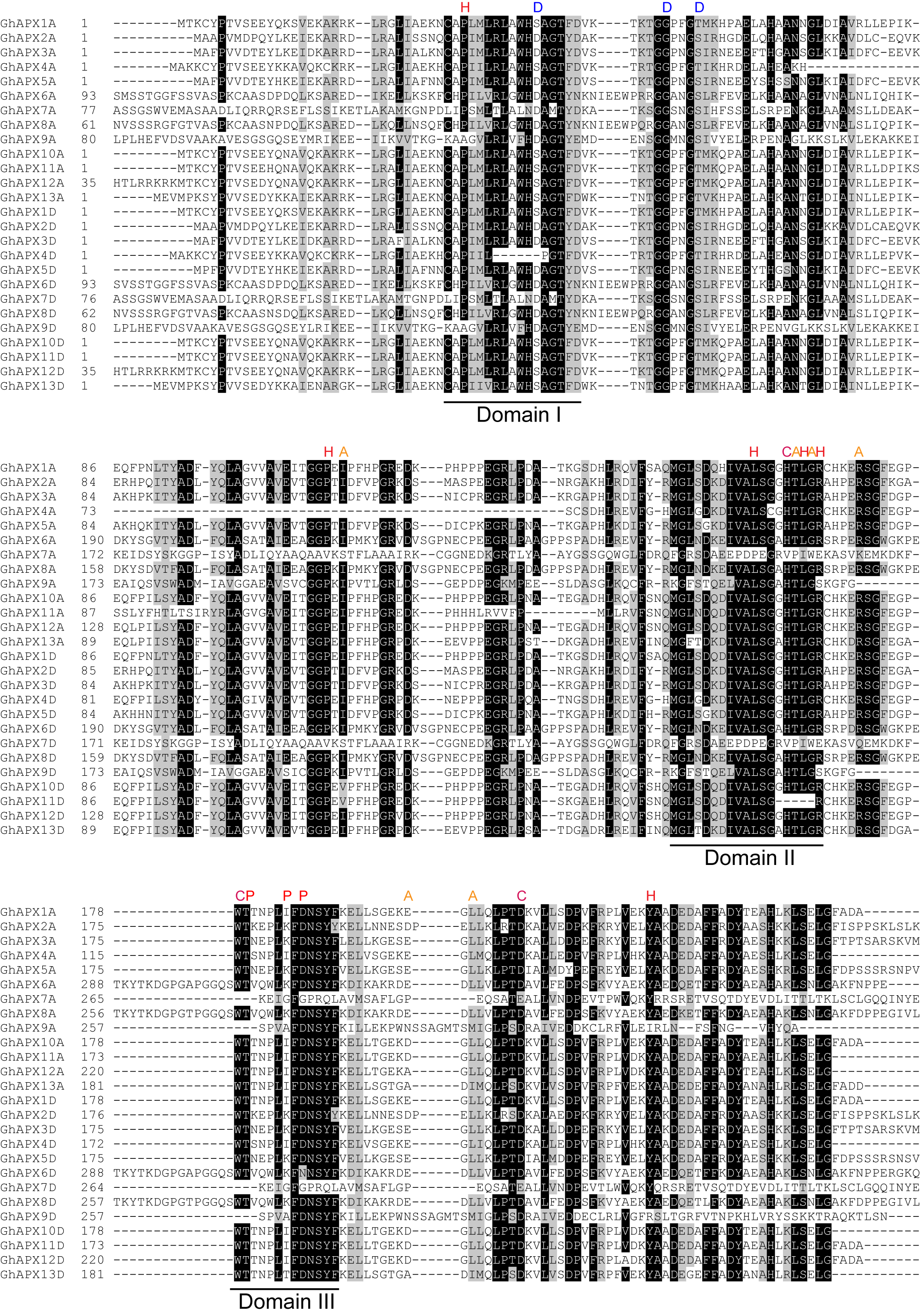

Supplement: Supplementary file 2 — Multiple sequence alignment of GhAPXs. Multiple sequence alignment was performed using the protein sequences of the 26 GhAPXs. Conserved domains I, II, and III were underlined. The label “H” marked the heme-binding amino acids, “D” the distal cation-binding sites, “A” ascorbate binding amino acids, “C” the amino acids involved in formation of the catalytic site, “P” the proximal cation biding sites (TIF 1830 KB) [file 438_2017_1413_MOESM2_ESM.tif]

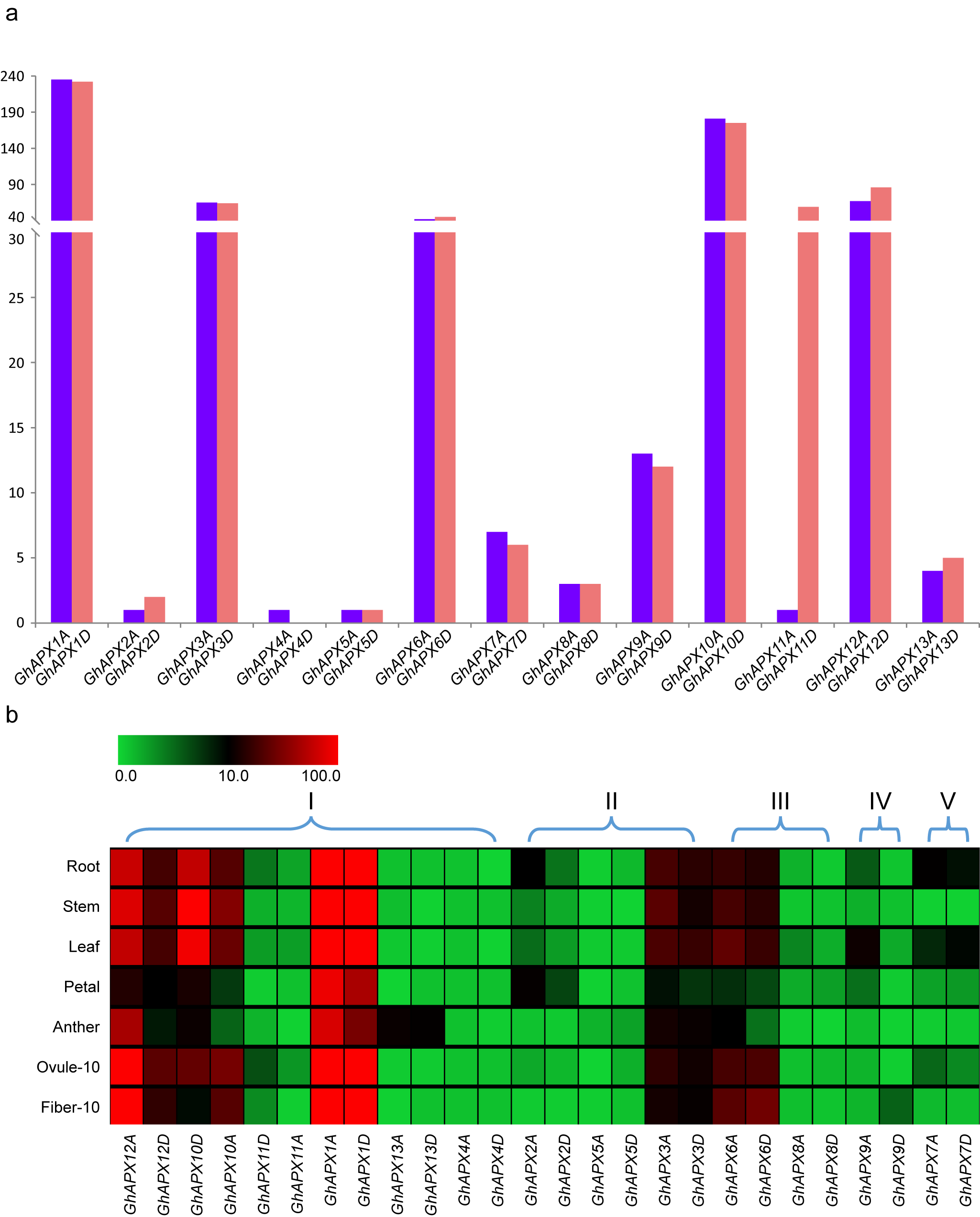

Supplement: Supplementary file 3 — Expression analysis of GhAPX genes using transcriptome and EST data. (a) The EST frequencies for each GhAPX gene were determined by screening over 0.3 million EST sequences downloaded from GenBank EST database. Higher EST hits numbers were considered to represent higher expression levels. Note the significant frequency difference between orthologs GhAPX11A and GhAPX11D, which is consistent with the qRT-PCR data shown in Fig. 4. (b) Heat map profiling of 26 GhAPX genes using RNA-seq data downloaded from genome database of G. hirsutum TM-1. The reads per kilobases per millionreads (RPKM) values were used to estimate the expression level of each GhAPX in different cotton tissues (TIF 1275 KB) [file 438_2017_1413_MOESM3_ESM.tif]
